# Duck production systems and highly pathogenic avian influenza H5N8 in France, 2016–2017

**DOI:** 10.1038/s41598-019-42607-x

**Published:** 2019-04-16

**Authors:** C. Guinat, J. Artois, A. Bronner, J. L. Guérin, M. Gilbert, M. C. Paul

**Affiliations:** 1 IHAP, Université de Toulouse, INRA, ENVT, Toulouse, France; 2 Université Libre de Bruxelles, Brussels, Belgium; 3 Direction Générale de l′Alimentation, Paris, France; 4 Fonds National de la Recherche Scientifique, Brussels, Belgium

## Abstract

In winter 2016–2017, Highly Pathogenic Avian Influenza (HPAI) H5N8 virus spread across Europe, causing unprecedented epizootics. France was massively affected, resulting in the culling of over 6 million poultry. Boosted regression tree (BRT) models were used to quantify the association between spatial risk factors and HPAI H5N8 infection in poultry holdings and to generate predictive maps for HPAI infection. Three datasets were combined to build the model: a dataset of the reported outbreaks in poultry, a dataset of the poultry holdings where the virus has not been reported and a set of relevant spatial risk factors, including poultry production and trade, and water bird habitat. Results identified key associations between the ‘*foie gras’* production systems and HPAI H5N8 risk of occurrence and indicate that strengthening surveillance of fattening duck production systems and making the transportation of fattening ducks more secure would be key priority options for HPAI prevention and control.

## Introduction

In winter 2016–2017, Highly Pathogenic Avian Influenza (HPAI) virus of the subtype A(H5N8) (clade 2.3.4.4.) caused severe and unprecedented epizootics across Europe, in terms of number of outbreaks in poultry and wild birds and number of affected countries^1,2^. Following its first official report of HPAI H5N8 outbreak in poultry on November 28, 2016, France was one of the most affected European countries by the disease. As of March 23, 2017, a total of 484 confirmed outbreaks in poultry and 52 cases in wild birds were reported^3^. Beginning in early 2017, in response to the emerging HPAI epizootic, French authorities implemented control measures, based on the application of pre-emptive culling and stringent movement restrictions. In addition to these control measures, French authorities in close collaboration with the poultry sector, increased farmers and veterinarians’ awareness and enhanced farm biosecurity, mainly focusing on the separation of poultry from wild birds, such as confining poultry and covering of feeding systems.

The literature on the spatial epidemiology of HPAI has been mainly related to the H5N1 subtype, with abundant research focusing on South East Asia^4^. Studies showed that the risk of HPAI infection in poultry was associated with anthropogenic factors, such as human population density and distance to roads, as well as with agro-environmental variables such as domestic waterfowl density and indicators of water presence^4^. In France, conditions favourable of HPAI H5N8 spread may somewhat differ from those reported in South East Asia due to difference in poultry production and environmental features. During the 2016–2017 epizootic, most of the outbreaks reported in France were located in southwest France, a region renowned for its ‘*foie gras*’ production, accounting for more than 70% of the world production. The fattening duck production system is characterised by three stages, often handled in different sites: rearing (1-day old ducklings reared indoor for 3 weeks), breeding (ducks reared outdoor with access to shelter for 9 weeks) and force-feeding (ducks force-fed indoor for up to 12 days).

So far, however, the role of these factors in the occurrence of HPAI H5N8 outbreaks has not been formally quantified, and the transmission pathways remain elusive. The objectives of this study were, therefore, to quantify the effect of several factors on the spatial distribution of outbreaks in the commercial poultry sector and to generate predictive risk maps for HPAI.

## Results

### Variables selection

**Figure 1:**
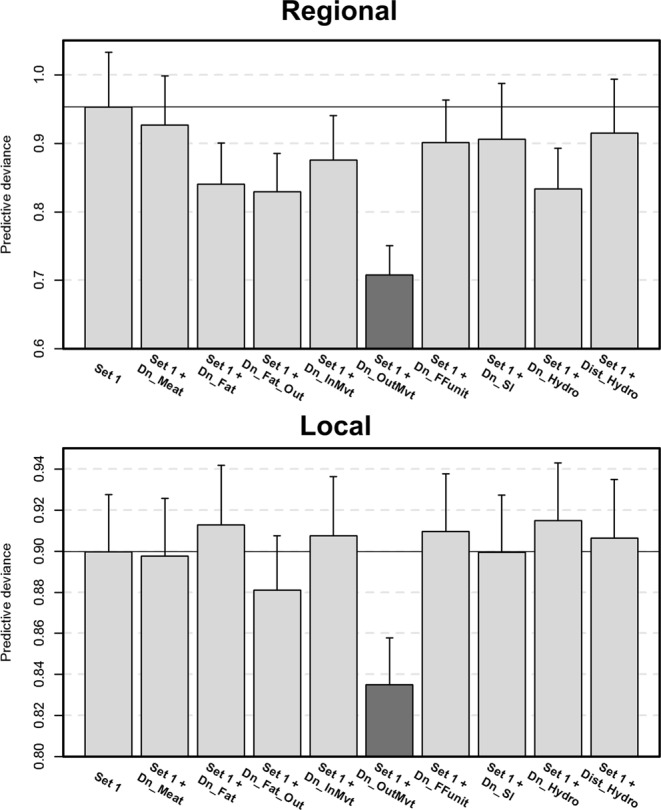
Representation of predictive deviance (average and standard error) of regional and local BRT models using spatial cross validation (CV). The bars in dark grey represent a significant difference in predictive deviance values between the Set 1 models and the Set 2 models using spatial CV. Dn_Meat: Density of holdings with ducks raised for meat per commune (/ha), Dn_Fat: Density of fattening duck holdings per commune (/ha), Dn_Fat_Out: Density of fattening duck holdings with outdoor access per commune (/ha), Dn_InMvt: Density of incoming fattening duck movements per commune (/ha), Dn_OutMvt: Density of outgoing fattening duck movements per commune (/ha), Dn_FFUnit: Density of force-feeding units per commune (/ha), Dn_Sl: Density of poultry slaughter houses per commune (/ha), Dn_Hydro: Density of waterways per commune (/ha), Dist_Hydro: Distance between the commune centroids and the closest water bodies (km).

The predictive deviances of the regional and local models for the different predictor variables and the spatial cross-validation (CV) method are shown in Fig. 1. The final BRT models have average correlation coefficients between the predicted and the observed response on test sets estimated at 0.574 (±0.011) for the regional model and 0.65 (±0.008) for the local model. For the regional and local model (Fig. 1), the combination of Set 1 and the density of outgoing movements did result in a better model than Set 1 alone using spatial CV. As a result, this variable was kept in the final local BRT model. Since results from the regional and the local models are similar, results from the local model only were presented here for clarity reasons. Predictive deviances for the standard CV are presented in Supplementary Fig. S1. The optimal number of trees and the number of predictor variables selected in the standard CV approach were higher than in the spatial CV approach. Indeed, seven and two predictor variables were selected with the standard CV method for the regional and the local models respectively, while only one predictor variable was selected with the spatial CV method for both spatial scales.

### Model outputs

**Figure 2:**
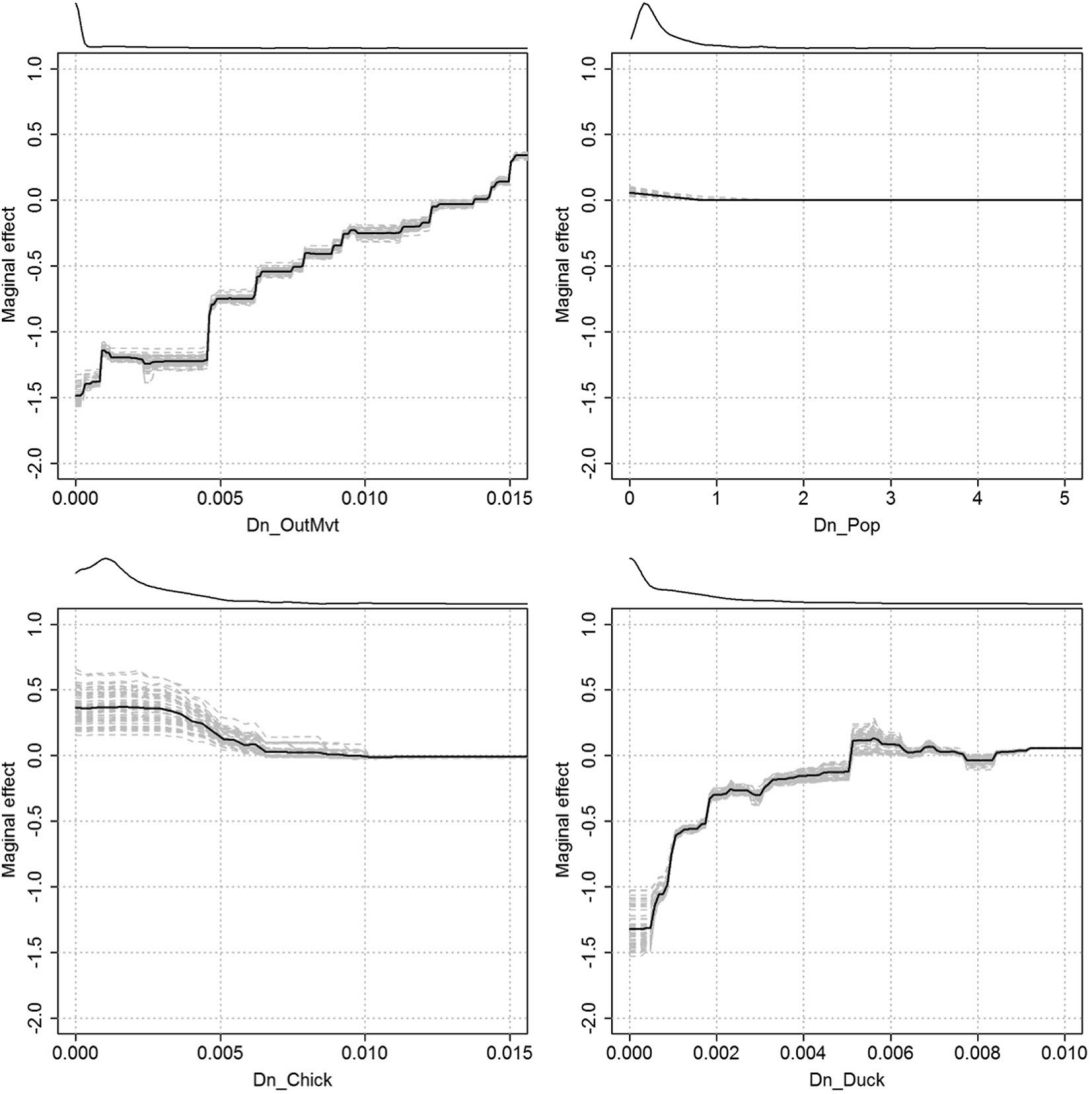
BRT profiles of the four predictor variables for the final local BRT models. The grey dashed lines represent the estimate for each bootstrap and the black dashed line is the mean over all bootstraps. The lines at the top of plots show the distribution of data of the variable on the X-axis. The variable Dn_Pop is log-transformed in the profile. Dn_OutMvt: Density of outgoing fattening duck movements per commune (/ha), Dn_Duck: Density of duck holdings per commune (/ha), Dn_Pop: Density of human population per commune (/ha), Dn_Chick: Density of chicken holdings per commune (/ha).

The BRT profiles of the four predictor variables for the final local BRT model are shown in Fig. 2. The proportion of infected holdings increased with the density of outgoing movements of fattening ducks up to a value of 0.015 movements per hectare (ha). Then, the proportion of infected holdings increased with the density of duck holdings up to a value of 0.005 per ha. The proportion decreased with the density of human population and the density of chicken holdings.

**Figure 3:**
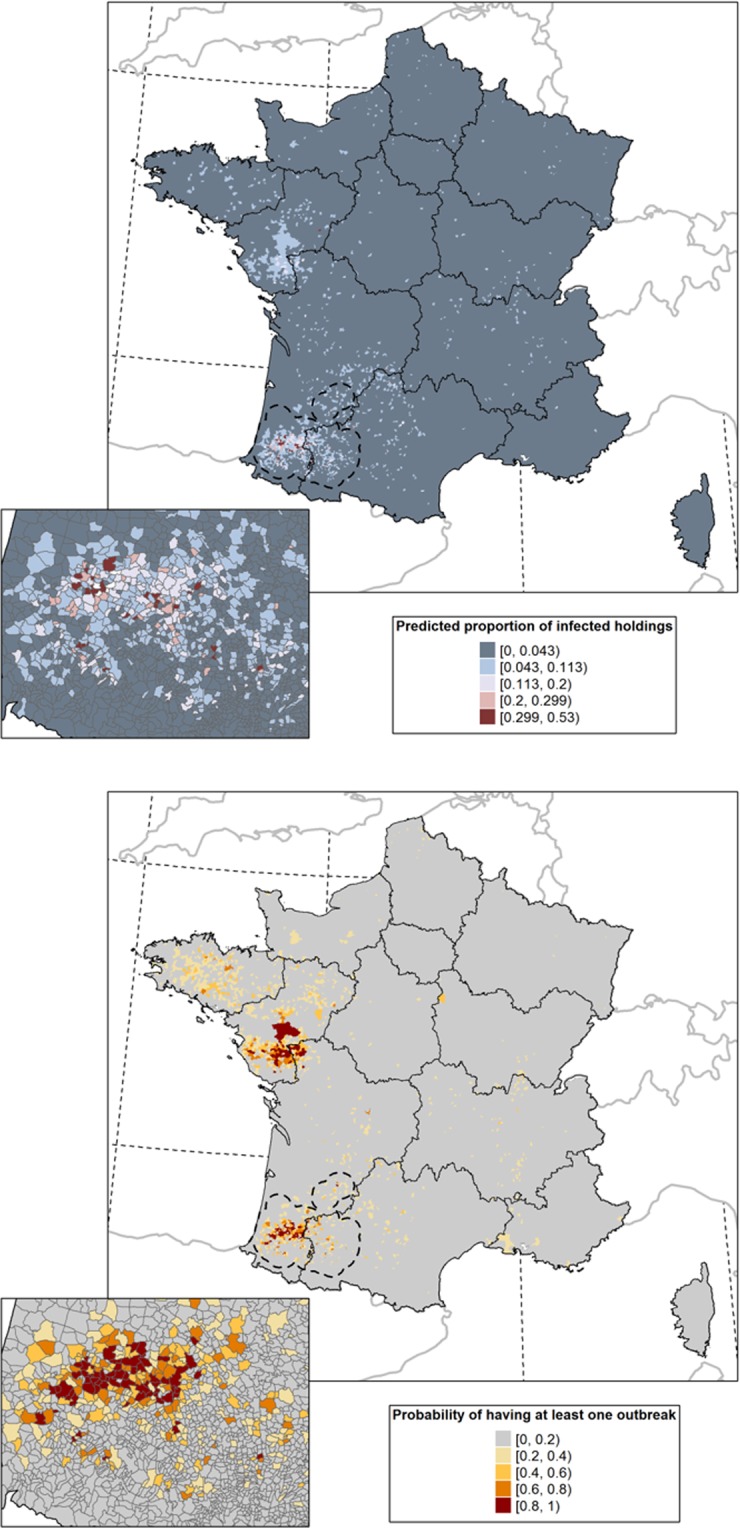
Predicted mean proportion of HPAI H5N8 infected holdings and probability of having at least one HPAI H5N8 infected poultry holding in the commune for the final local BRT model. The dashed black line represents the local scale.

The HPAI H5N8 prediction map for the final local BRT model is shown in Fig. 3. As expected, high proportions of infected holdings were predicted in southwest France. The model also predicted that the proportion of infected holdings was moderate in northwest France where HPAI H5N8 was introduced but did not persist during the 2016–2017 epizootics. The uncertainty maps associated with the predictions are presented in Supplementary Fig. S2.

## Discussion

This study allowed spatial predictor variables related to poultry production to be ranked according to their importance on the spatial distribution of HPAI H5N8 outbreaks in France during the 2016–2017 epizootic. The density of outgoing movements of fattening ducks (See Supplementary Fig. S3) was selected in both analyses, at the regional and local scales. This demonstrates that this variable was not only representative of regional differences related to fattening duck production (See Supplementary Fig. S3), but rather would explain outbreaks occurrence in southwest France. Fattening ducks are bred in large flocks of several thousand for up to 12 weeks, which are then frequently divided into small batches of hundreds of ducks to be moved to force-feeding units for 12 days. Thus, the short production cycle and the small size of force-feeding units facilitates direct and indirect contacts between fattening duck flocks along transport networks. Poultry trade data has been rarely integrated in disease spatial modelling^4^, with road density and distance to road generally used as surrogates of poultry movements. Thus, the results highlight the importance of considering poultry trade data especially if one considers its contribution to HPAI occurrence in the present study. While a straightforward interpretation is still lacking regarding the higher contribution of outgoing movements compared to incoming movements (See Supplementary Fig. S3), this is likely explained by contact mechanisms related to human activities that have been identified before movements of flocks, such as catching teams who assisted in the catching of ducks before being sent to force-feeding units and who are frequently moving between holdings^5^. This could also be linked to the cleaning and disinfection processes that represent a major challenge to eliminate the virus in empty lorries before returning to duck holdings^6^. In addition, this could reflect a higher probability of detection in duck flocks before movement, although data suggest that active surveillance of duck flocks before movements represented only a limited part of the overall number of outbreaks reported. Although adding information about fattening duck movements improved model predictability, the Set1 model trained here with only three predictor variables (human population density, chicken holding density and duck holding density) allowed predicting the broad-scale spatial distribution of HPAI H5N8 outbreaks in France with relatively good results. First, as with a previous study^7^, the duck holding density was positively associated with the proportion of infected holdings by HPAI H5N8. The chicken holding density (See Supplementary Fig. S3) was negatively associated with the proportion of infected holdings, which may be explained by the hypothesis that HPAI H5N8 virus would be less adapted to chicken than ducks^8^. While human population density was often reported as one of the top predictors for other HPAI H5 viruses’ infection^4^, it was here negatively associated with the proportion of infected holdings but with a low relative contribution in BRT. This variable is often used as a proxy of higher likelihood of outbreak detection and of higher likelihood of transmission through poultry-related trade^4^. Here it may rather reflect the area of southwest France where HPAI H5N8 emerged, with relatively low human demographics (See Supplementary Fig. S3), at regional and local scales.

In all cases, the predictive deviance (the error) of the final BRT models increased when using spatial CV instead of standard CV, which is in line with the hypothesis that standard CV may be overfitting the data^9^ due to the lack of independence between training and testing data. Overall, the spatial CV approach allows selecting less complex BRT models (which might be more easily transferable over the landscape) based on a lower number of predictor variables and of trees compared to the standard CV approach. Despite significant variables identified in the final BRT models, some variability remained unexplained. First, the results might be influenced by the variability between the poultry holding census data used in this study and the number of holdings that were actually active (i.e. filled with poultry) at the time of the epizootics. Moreover, variables related to water presence were used as surrogate estimates of the wild bird distribution, while having quality data on wild bird migration flyways would improve the predictions. Here, only movements of fattening duck flocks were integrated, since movement records of vehicles (with regards to catching, rendering and slaughtering processes) were not available. Also, variables should be considered in conjunction with other factors, such as conditions affecting virus survival in the environment. However, uncertainty maps allowed to identify areas where the model predictions were less accurate, thus where predictions should be interpreted with caution, such as those made at long distances from points of presence.

Recently developed for predicting the distributions of organisms in ecological studies^10,11^, BRT approaches have been shown to out-perform the more traditional regression approaches in term of predictive performance^10^. They have therefore recently been a focus of attention in the field of health epidemiology, providing accurate spatial risk predictions of major infectious diseases, including HPAI^12,13^, Ebola^14^ or Dengue^15^. They can fit complex nonlinear relationships and automatically model interactions between predictors. BRT models provide visual and intuitive outputs; such as profiles of the effect of each predictor variable on the response variable over the range of its values. This allows determining the ranges of values of the predictor variables over which the effect was most important, which can support the design of control strategies. In addition, the BRT models performance comparison approach used here for the selection of predictor variables allowed us to decrease the number of predictor variables that were used in the final models. While the density of outgoing fattening duck movements identified in the final BRT models was correlated to the density of duck holdings in Set 1 which could obscure the relative contribution of these two variables, the BRT models performance comparison allowed us to conclude that the density of outgoing fattening duck movements and variables of Set 1 had independent contributions in the final model.

In conclusion, the results highlight that the trade-related transport of fattening ducks has contributed to HPAI H5N8 outbreak occurrence in France during the 2016–2017 epizootic. This is especially true considering the clustered distribution of outbreaks in southwest France, while the entire country was exposed to HPAI H5N8 due to the proximity of severely affected neighboring countries such as Germany and Switzerland^1^. These findings are important since they suggest that preventing future poultry infections with HPAI H5N8 virus (clade 2.3.4.4.) should be focusing, among other measures, on strengthening surveillance of duck production systems and on making transportation of ducks more secure, rather than changing holding characteristics (such as the presence of outdoor access). As a result, major changes have been implemented by the French authorities, strongly supported by the fattening duck sector, to enhance biosecurity on farms and along the transport of ducks, as well as to reinforce disease surveillance strategies. Lorries are now required to comply with reviewed procedures of transport and guidelines of cleaning and disinfection of cages to improve effectiveness in preventing the spread of HPAI virus. During the high risk period between December and March, diagnostic tests are also performed on duck flocks before being moved^16^. Assuming similarities in production systems, the outcomes could also contribute to better understand the spatial distribution of HPAI H5N8 outbreaks in Europe, where other large duck-producer countries, such as Bulgaria and Hungary^17–19^, were severely impacted by the disease. The prediction risk map generated here gives a more refined picture of HPAI H5N8 risk which could be used to inform policy and resource allocation decisions. This study provides important insights on the factors influencing the spatial distribution of HPAI H5N8 outbreaks at a national scale. However, results also argue the need for future studies to further explore the effect of poultry flock movements and between-farm connectivity, as well as other farm-level characteristics such as biosecurity practices on the risk of HPAI H5N8 outbreak occurrence.

## Methods

### Data collection

#### H5N8 poultry outbreaks

**Figure 4:**
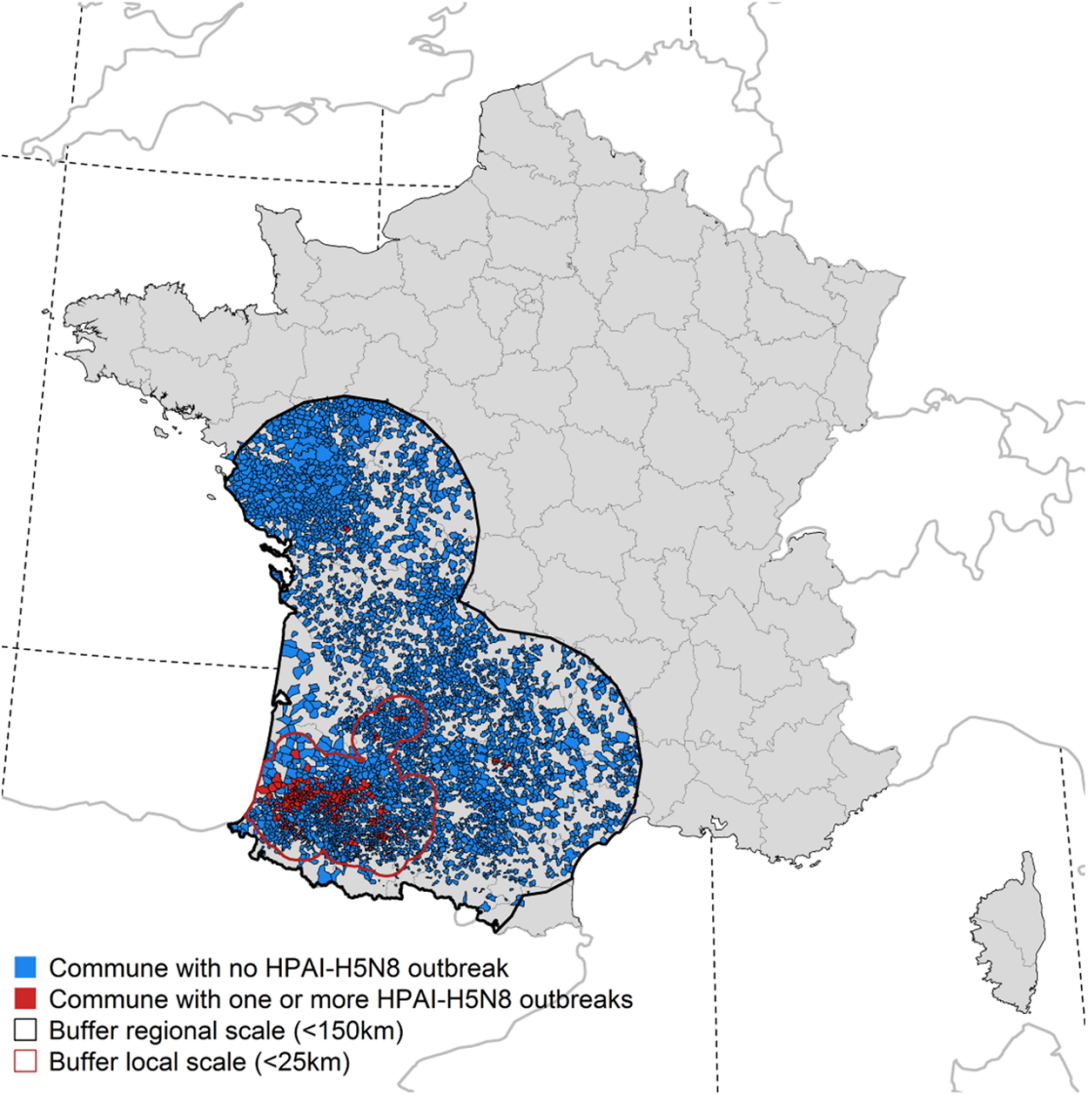
Geographical distribution of the positive (with one or more HPAI H5N8 outbreaks in poultry) and negative (with no HPAI H5N8 outbreaks in poultry) communes included in the BRT models at the regional and local scales. Communes in grey colour were not considered and were those without poultry.

Data regarding all confirmed HPAI H5N8 outbreaks (n = 478) in commercial poultry holdings in France were obtained through the French Ministry of Agriculture (DGAl). A confirmed outbreak was defined as the detection of at least one laboratory-confirmed HPAI H5N8/Nx infected animal (by virus isolation or PCR) in a commercial poultry flock. Outbreak data were grouped at commune (smallest administrative unit in France, with a median surface of 10 km2) level over the period of November 2016 to March 2017, and geo-referenced using the commune centroid coordinates obtained from GEOFLA® (http://professionnels.ign.fr/geofla). While communes with one or more HPAI H5N8 outbreaks were considered as positive cases, pseudo-negative cases were generated based on two conditions: (i) having a number of poultry holdings >1 to exclude communes without commercial poultry production, and (ii) no HPAI H5N8 outbreaks had been reported (Fig. 4).

#### Spatial predictor variables

Twelve variables, mainly related to poultry production and water bird habitat were considered for their potential influence on the spatial distribution of HPAI H5N8 outbreaks (Table 1). Set 1 included the three following variables containing general information on hosts: the densities of human population, of chicken holdings and of duck holdings per commune. The density of chicken and duck commercial holdings per commune was computed from the DGAl and the organisation of fattening duck producers (CIFOG) databases, which registers all producers of the poultry and the fattening duck sector, respectively. In addition, the human population density per commune generated from GEOFLA® (http://professionnels.ign.fr/geofla) was included as a proxy variable for surveillance bias and to account for any anthropogenic transmission mechanisms. Set 2 included the nine following variables containing detailed characteristics of the duck production systems: the densities of holdings raised for meat, of fattening duck holdings, of fattening duck holdings with outdoor access, of incoming and of outgoing fattening duck movements, of force-feeding units, of poultry slaughter houses, of waterways per commune, and the distance between the commune centroids and the closest water bodies. The density of holdings with ducks raised for meat, of fattening duck holdings and of duck holdings with outdoor access per commune were computed by commune from the DGAl and CIFOG databases. The density of outgoing (from the breeding stage) and the density of incoming (to the force-feeding stage) fattening duck movements per commune was computed from the CIFOG database over the period of October 2016 to February 2017. The list of force-feeding units and of poultry slaughter houses per commune were obtained through the DGAl, from which densities were also computed for each commune. Data on the spatial distribution of wild bird species is generally difficult to obtain at high resolution, with surveillance efforts varying strongly according to the season and to the geographical areas. Therefore, indicators of water presence were used as proxy variables to quantify the possible association between wild birds’ habitats and the distribution of HPAI H5N8 outbreaks. The density of waterways (i.e., rivers, streams, canals, etc. with length >20 m per ha) and the distance between the commune centroids and the closest water bodies (i.e., lakes, swamps, reservoirs, floods, etc. with area >25 ha) were thus generated from BD CARTO® (http://professionnels.ign.fr/bdcarto). Climatic factors were not tested in this analysis, because HPAI outbreaks have been reported in Europe, Asia and Africa over a large range of temperatures and environmental conditions^4^, which supports the hypothesis that climate might not spatially constrain the occurrence of outbreaks. ArcGIS 10.4 software (ESRI Inc.) and R software version 3.4.2^20^ were used for manipulation of spatial data.

**Table 1:** Predictor variables used in the BRT models.

Set	Variables	Abbreviation	Source*
1	Density of human population per commune (/ha)	Dn_Pop	IGN
	Density of chicken holdings per commune (/ha)	Dn_Chick	DGAl
	Density of duck holdings per commune (/ha)	Dn_Duck	CIFOG, DGAl
2	Density of holdings with ducks raised for meat per commune (/ha)	Dn_Meat	DGAl
	Density of fattening duck holdings per commune (/ha)	Dn_Fat	CIFOG
	Density of fattening duck holdings with outdoor access per commune (/ha)	Dn_Fat_Out	CIFOG
	Density of incoming fattening duck movements per commune (/ha)	Dn_InMvt	CIFOG
	Density of outgoing fattening duck movements per commune (/ha)	Dn_OutMvt	CIFOG
	Density of force-feeding units per commune (/ha)	Dn_FFunit	CIFOG
	Density of poultry slaughter houses per commune (/ha)	Dn_Sl	DGAl
	Density of waterways per commune (/ha)	Dn_Hydro	IGN
	Distance between the commune centroids and the closest water bodies (km)	Dist_Hydro	IGN

^*^IGN: Institut National de l′Information Géographique et Forestière.

DGAl: Direction Générale de l′Alimentation of the French Ministry of Agriculture.

CIFOG: Comité Interprofessionnel des Palmipèdes à Foie Gras.

#### Spatial scale

Southwest and northwest France are two main regions for duck production, although most of the outbreaks were reported in fattening duck holdings located in southwest France (Fig. 4). Considering the different types of production in these two regions, two spatial scales were used to distinctly analyse the risk factors explaining the distribution of outbreaks over these two production basins and those that shape the distribution of outbreaks within the infected area (i.e. southwest). The communes being at a maximum distance <25 km of any positive (for the local scale), or being at a maximum distance <150 km of any positive (for the regional scale) were selected as spatial domains of analysis (Fig. 4).

### Data analysis

#### Boosted regression tree models

Poisson BRT models^21–23^ were used to quantify the effect of spatial risk factors on the proportion of infected holdings by HPAI H5N8 virus and to map the resulting predicted risk. The models were developed using the number of infected poultry holdings per commune as the dependent variable with an offset term corresponding to the total number of poultry holdings per commune. BRT models are based on an iterative procedure whereby a regression tree is fitted onto a dependent variable, followed by estimating residuals and then fitting new trees on residuals that best reduces the loss function (deviance), updating predictions and residuals. The procedure is repeated iteratively until an optimum in predicted deviance (CV) is reached. BRT models were run with the following parameters: a tree complexity of 3, a learning rate of 0.003 and a bag fraction of 0.75. BRT models are set and evaluated using 4-fold CV procedure to determine the optimal number of trees that maximises the predictive capacity and the generalization performance of the models^21^, while limiting model overfitting (set with an initial number of trees of 100 and a step size of 50 trees). In the standard CV procedure, BRT partitions the data into four random folds, with three folds used as a training set to build the model and one fold used as a test set to evaluate its predictive capacity. Here, the HPAI outbreaks are clustered among poultry farms and the spatial predictor variables are strongly regionalised. The resulting sets are therefore not necessarily independent because the selection of data for the training and test sets is made at random. In such case, spatially correlated observations could be split over the test or training set during the CV procedure and violate the independence assumption of the test/training set. In case the test set is non-independent of the training set, the procedure to determine the optimal number of trees will fail and the optimal number of trees selected to adjust a BRT will be overestimated. Therefore, spatial CV was implemented to decrease the probability that two observations aggregated in space were split between the test and training set and to measure the generalization performance of the models. The spatial CV uses the ‘block’ method to partition the dataset into four spatial folds based on the lines of latitude and longitude that divide occurrence localities as equally as possible^9,24^. Finally, the procedure was repeated 50 times to account for sources of uncertainty in data splitting.

The generalization performance of the BRT models was assessed by calculating the predictive deviance, i.e., y – p_f_, where y is the dependant variable from testing dataset and p_f_ the fitted probability. BRT profiles were generated to determine the relationship between the predictor variable and the proportion of infected holdings. To facilitate results interpretation, the predicted proportion of infected poultry holdings was converted into a probability of having at least one outbreak in the commune estimated with a Binomial distribution as following, P(X > 0) = 1 − (1-p)^n^, where n is the number of poultry holdings in the commune and p is the proportion of infected holdings predicted by the Poisson BRT model. The mean predicted probability of having at least one infected poultry holding per commune was generated and mapped over the whole of France. To check the uncertainty of prediction, the standard deviation of the predictions was also mapped. Models were run in R software version 3.4.2^20^ using dismo package^21^. The data generated and analysed during the current study are available from the corresponding author on reasonable request.

#### Variables selection

A comparison of the BRT models performance was used to reduce the total number of predictor variables in order to deal with multi-collinearity among predictor variables related to duck production (i.e. the coefficient correlations ranged from 0.52 to 0.78 between the density of duck holdings and the densities of holdings with ducks raised for meat, of fattening duck holdings, of fattening duck holdings with outdoor access, of force-feeding units, of incoming and outgoing fattening duck movements) and to limit the model overfitting. Indeed, multi-collinearity among predictor variables may increase variance in BRT predictions and results fitted over small data sets^21^. First, a Set 1 model was fitted with only three variables (Set 1), which contained general information on hosts (Table 1). Second, nine variables (Set 2), containing detailed characteristics of the duck production systems, were individually added to the Set 1 model and the predictive deviances were compared between the nine intermediate models and the Set 1 model. Third, a final model was built with the three variables of Set 1 and variables of the intermediate models that decreased the predictive deviance of the Set 1 model based on a threshold criterion (i.e. the reduction in predictive performance reaches twice the value of its standard error^21,25^). Finally, the predictive deviance of the final model was compared to those of the Set 1 and intermediate models. Such forward approach did ensure that a part of variation in the dependent variable was explained by the predictor variables of Set 2 independently, at least partially, of the Set 1 information in the final model. Ultimately, the generalization performance of the final BRT models was also assessed by calculating the Pearson correlation coefficient between the predicted and the observed response on test sets.

## Supplementary information

Supplementary Figures
