# Supplementary material for: Duck production systems and highly pathogenic avian influenza H5N8 in France, 2016–2017
Source: Sci Rep. 2019 Apr 16;9:6177. doi: 10.1038/s41598-019-42607-x (PMC6467959; doi:10.1038/s41598-019-42607-x)
Supplement: Supplementary file 1 — Supplementary Figures [file 41598_2019_42607_MOESM1_ESM.pdf]

Duck production systems and highly pathogenic avian influenza H5N8 in France, 2016-2017

C. Guinat[1,2,*], J. Artois[3], A. Bronner[4], J.L. Guérin[1,2], M. Gilbert[3,5], M.C. Paul[1,2]

[1]École Nationale Vétérinaire de Toulouse, Toulouse, France

[2]Institut National de la Recherche Agronomique, Toulouse, France

[3]Université Libre de Bruxelles, Brussels, Belgium

[4]Direction Générale de l'Alimentation, Paris, France

[5]Fonds National de la Recherche Scientifique, Brussels, Belgium

*c.guinat@envt.fr

Supplementary Figures

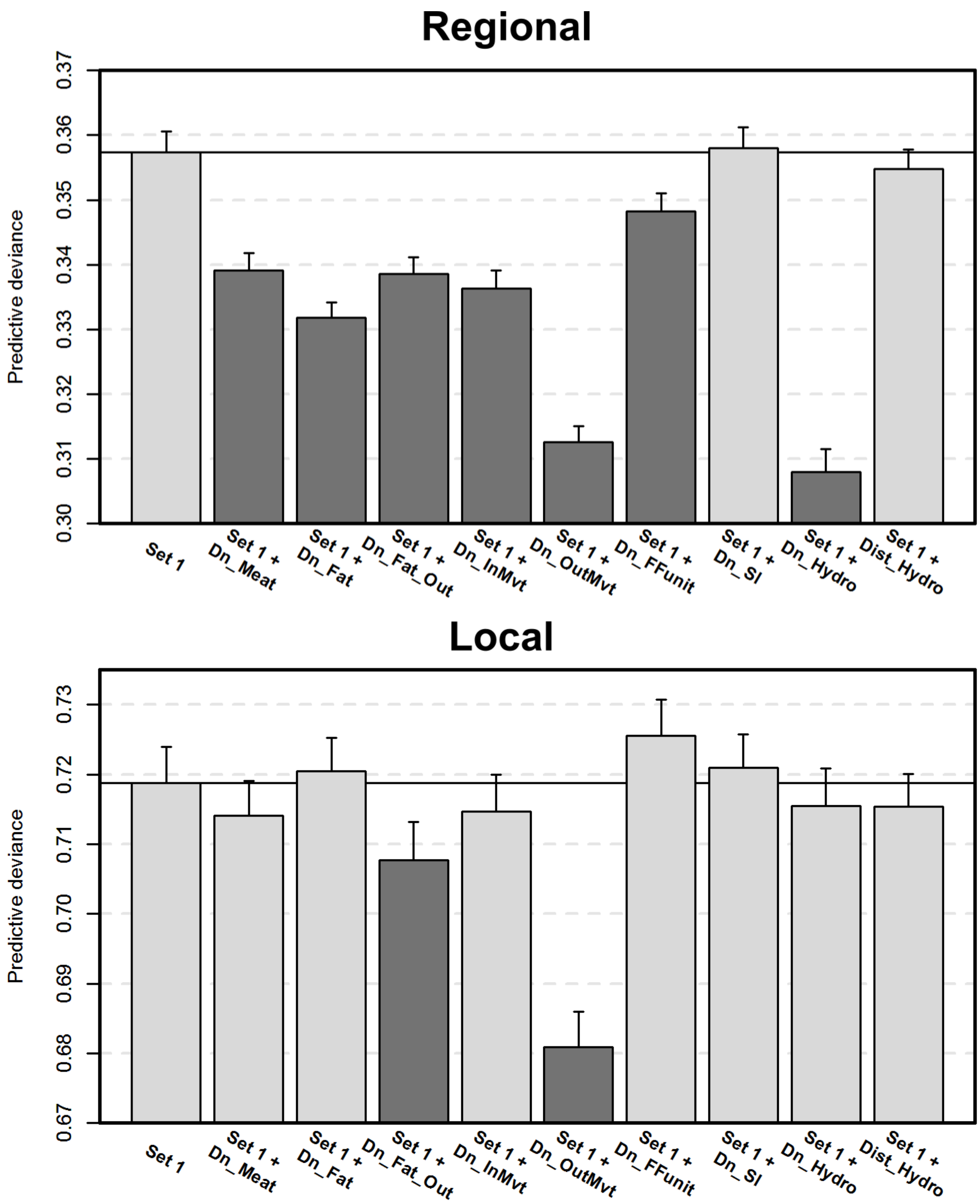

Fig. S1. Representation of predictive deviance (average and standard error) of regional and local BRT models using standard cross validation (CV). The bars in dark grey represent a significant difference in predictive deviance values between the Set 1 models and the Set 2 models using standard CV. Dn_Meat: Density of holdings with ducks raised for meat per commune (/ha), Dn_Fat: Density of fattening duck holdings per commune (/ha), Dn_Fat_Out: Density of fattening duck holdings with outdoor access per commune (/ha), Dn_InMvt:

Density of incoming fattening duck movements per commune (/ha), Dn_OutMvt: Density of outgoing fattening duck movements per commune (/ha), Dn_FFUnit: Density of force-feeding units per commune (/ha), Dn_Sl: Density of poultry slaughter houses per commune (/ha), Dn_Hydro: Density of waterways per commune (/ha), Dist_Hydro: Distance between the commune centroids and the closest water bodies (km)

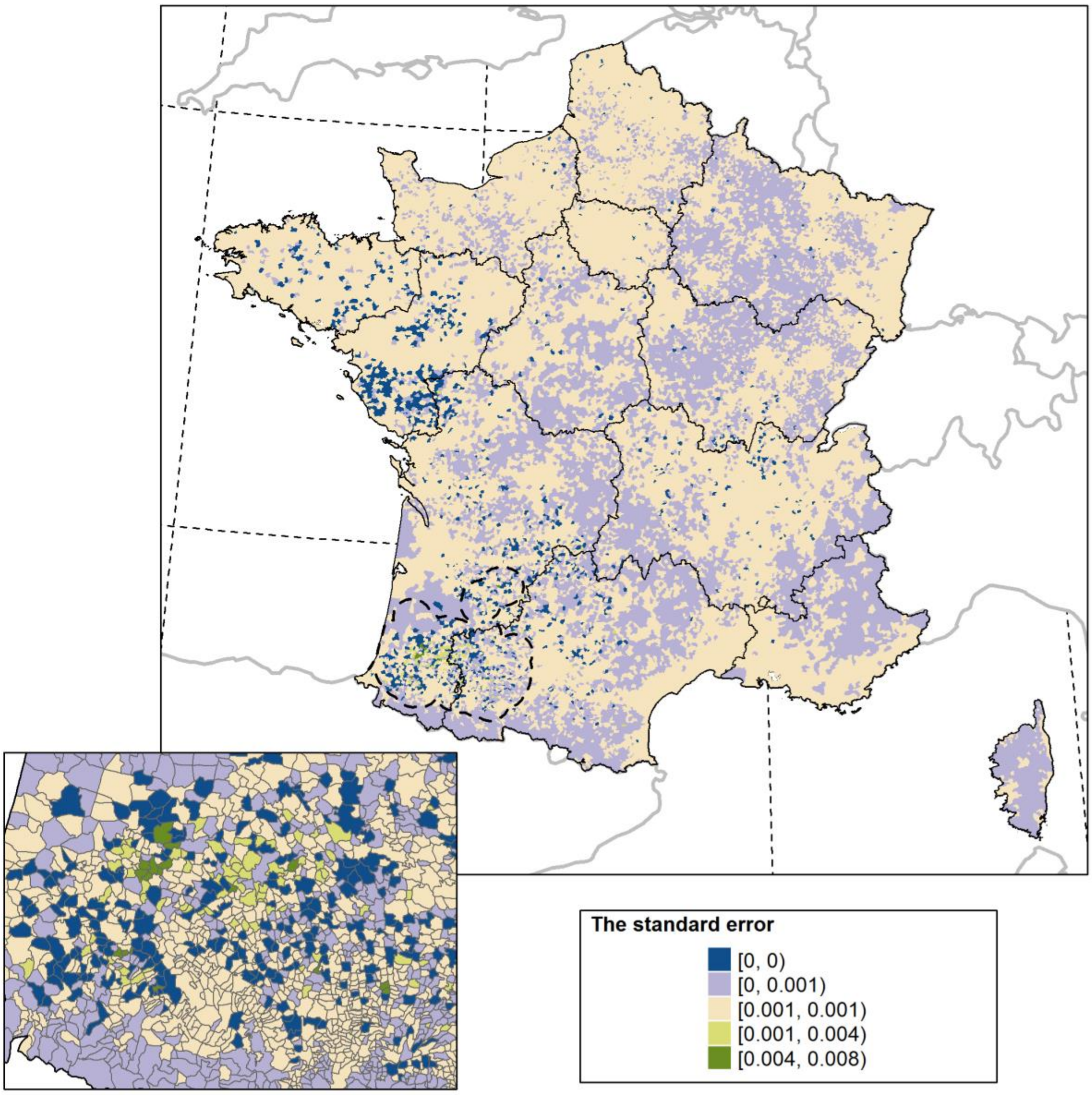

Fig. S2. Predicted uncertainty (standard error) of the probability of having at least one HPAI H5N8 infected poultry holding in the commune for the final local BRT model. The dashed black line represents the local scale.

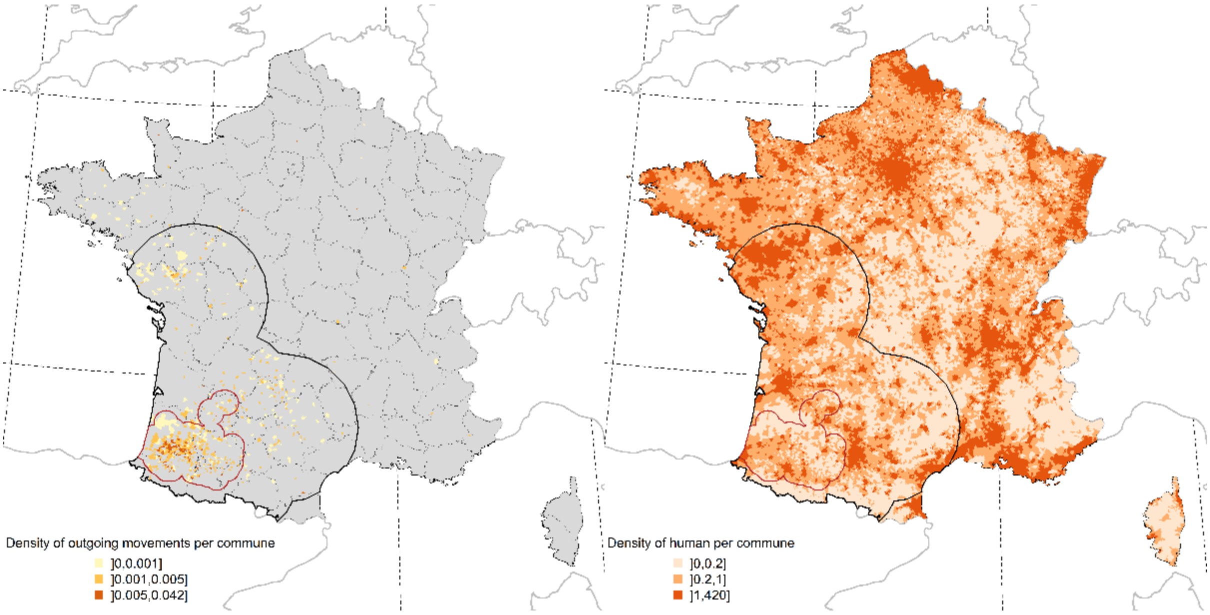
Density of outgoing movements per commune
]0,0.001]
]0.001,0.005]
]0.005,0.042]
Density of human per commune
]0,0.2]
]0.2,1]
]1,420]

28

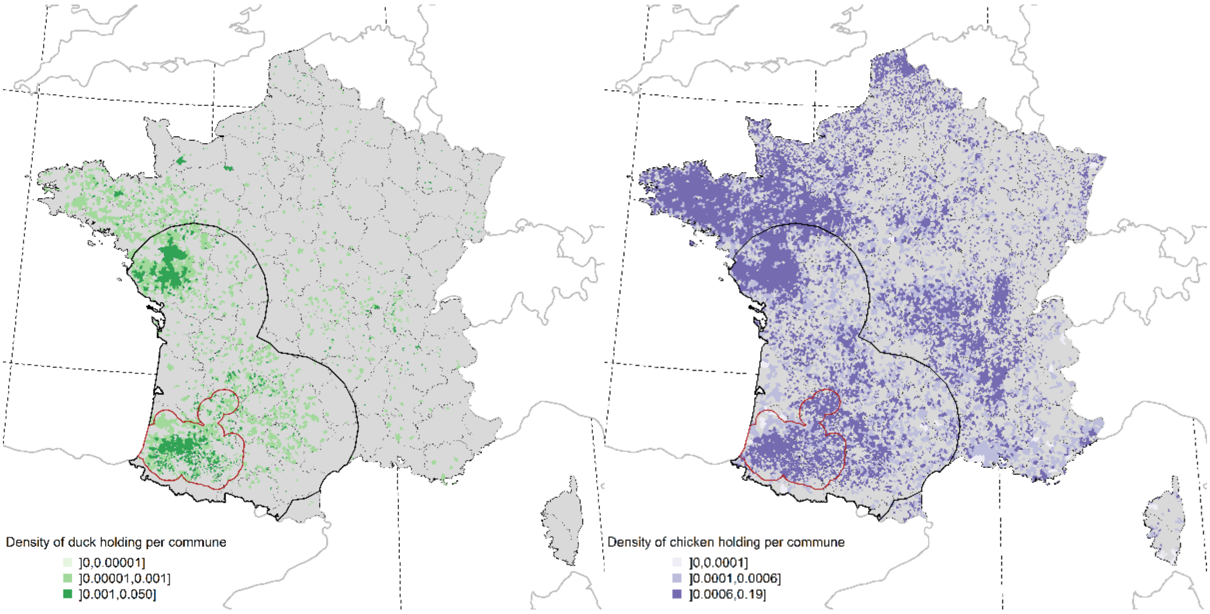
Density of duck holding per commune
]0,0.00001]
]0.00001,0.001]
]0.001,0.050]
Density of chicken holding per commune
]0,0.0001]
]0.0001,0.0006]
]0.0006,0.19]

29

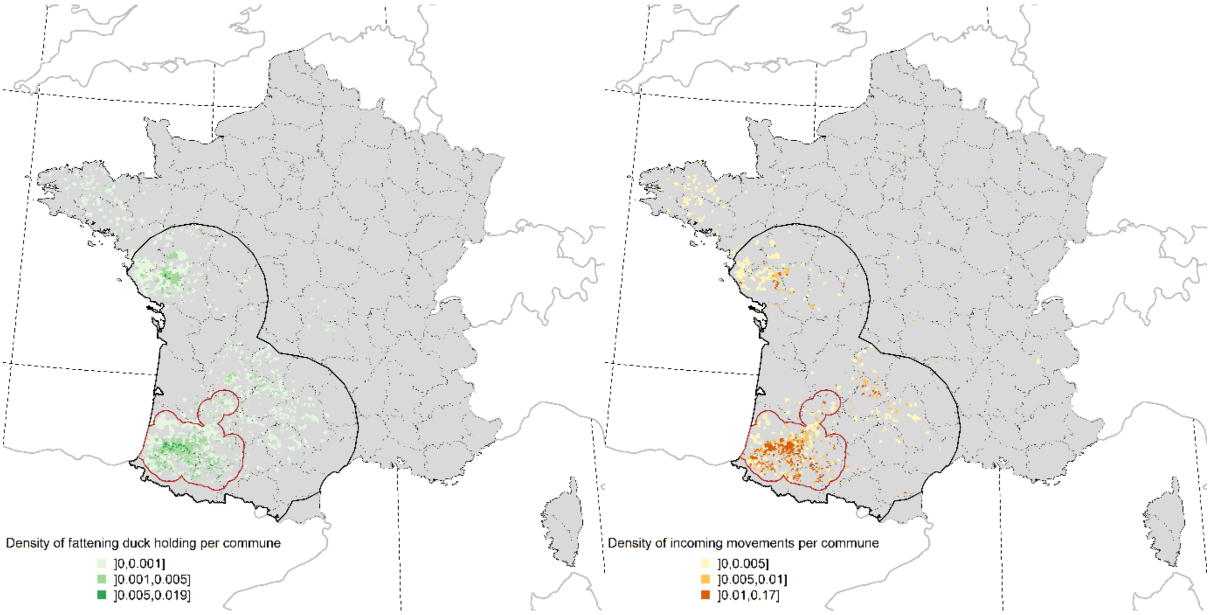

Fig. S3. Geographical distribution of predictors variables used in the study. The black and red lines represent the regional and local scales, respectively.
